# 

**Published:** 1950-07

**Authors:** 


### CONTENTS

| | Page |
|---|---|
| The Work of a Department of Industrial Health. By R. C. BROWNE, D.M. M.R.C.P. | 67 |
| The General Use of Histamine Antagonists. By ROBERT P. WARIN, M.D., M.R.C.P. | 75 |
| Impressions of American Rheumatology and Recent Advances in "Rheumocrinology". By G. D. KERSLEY, T.D., M.D., M.A., F.R.C.P. | 82 |
| The Value of X-Ray Therapy for Metastases in Bone from Carcinoma of the Breast. By SYDNEY CURWEN, D.M.R.T. | 88 |
| Editorial Note | 92 |
| Obituary | 93 |
| Reviews of Books | 94 |
| Meetings of Societies | 96 |
| Local Medical Notes | 100 |

